# Ae index is an independent predictor of kidney stone recurrence in overweight and obese patients

**DOI:** 10.1186/s12894-023-01321-7

**Published:** 2023-09-23

**Authors:** Kaiguo Xia, Yuexian Xu, Qiao Qi, Jiashan Pan, Rui Yao, Qingfeng Huang, Zongyao Hao

**Affiliations:** 1 Department of Urology, The First Affiliated Hospital of Anhui Medical University, Hefei, 230022 China; 2 Institute of Urology, Anhui Medical University, Hefei, 230022 Anhui China; 3 Anhui Province Key Laboratory of Genitourinary Diseases, Anhui Medical University, Hefei, Anhui 230022 China

**Keywords:** Apo B, eGFR, Ae index, Kidney stone recurrence, Overweight, Obesity

## Abstract

**Background:**

Finding some convenient and economical indicators to initially screen overweight and obese patients at high risk of kidney stone recurrence can help them prevent stone recurrence with lower medical cost. The purpose of this article is to determine the clinical value of Ae index (Apo B × 1000/eGFR) as an independent predictor for kidney stone recurrence in overweight and obese populations.

**Methods:**

We queried the electronic medical records of patients with kidney stone operated at our hospital from March 2016 to March 2022, and selected BMI ≥ 25 kg/m^2^ as the study population and divided the patients into stone recurrence group and non-recurrence group. Relevant parameters of routine blood and biochemical test, glycated serum protein (GSP), and history of hypertension and hyperglycemia were collected. Then the Chi-square test, independent samples t-test or Wilcoxon rank-sum test were used to calculate the differences between the two groups of data. Next, we performed univariate and multivariate logistic regression analysis to screen out the most significant variables Apo B and eGFR, and then we calculated the Ae index using the formula Apo B × 1000/eGFR, and analyzed the relationship between Ae index and kidney stone recurrence.

**Results:**

Univariate analysis found that Apo B (OR:8.376,95%CI:3.093–22.680), Creatinine (OR:1.012,95%CI:1.003–1.021), Cystatin C(OR:2.747,95%CI:1.369–5.508), LDL-C (OR:1.588,95%CI:1.182–2.134), TC (OR:1.543,95%CI:1.198–1.988) were positively associated, eGFR (OR:0.980,95%CI:0.970–0.991) was negatively associated with kidney stone recurrence. And multivariate logistic regression analysis suggested that Apo B (OR:11.028, 95%CI:3.917–31.047) and eGFR (OR:0.976, 95%CI:0.965–0.988) were the most significant factors. Then we calculated Ae index and analyzed it, the sensitivity was 74.26% and the specificity was 60.00%, higher than either individual variable. Its smoothed curve revealed a non-linear relationship between them with the inflection point of 9.16. And the OR on the left side of the inflection point was 1.574 (95% CI: 1.228–2.018), whereas the OR on the right side of the inflection point was 1.088 (95% CI: 1.007–1.177).

**Conclusions:**

Ae index is an easily calculated and obtained index that has some predictive value for kidney stone recurrence in overweight and obese patients, which is of interest.

**Supplementary Information:**

The online version contains supplementary material available at 10.1186/s12894-023-01321-7.

## Introduction

Kidney stone is a common disease in urology, which can cause urinary tract obstruction and affect kidney function in serious cases [1–4]. Currently, the incidence of kidney stone is increasing year by year, with about 7%-13% in North America, 5%-9% in Europe, 1%-5% in Asia, and 6.3% in China [5–7]. In addition, kidney stone is also known as a lifelong disease because of the high recurrence rate, and it has been reported that about half of the patients with kidney stone disease would experience a second attack, and more than 10% experience more recurrence [8, 9]. Siener R reported a 50% recurrence rate of kidney stone [10], and Zisman AL reported that the recurrence rates of kidney stone diseases at 2, 5,10, and 15 years were 11%, 20%, 31%, and 39% [11]. Compared to other urological diseases, kidney stone diseases are more expensive to treat, and more than $5 billion was spent annually on kidney stone in the United States [12]. The recurrence of stones not only increases the pain caused by the disease but also leads to secondary treatment and increases the financial burden of the patient. Therefore, it is important to prevent the recurrence of kidney stone.

There are many factors that influence the recurrence of kidney stone, both in relation to the patient's race, family history, age, and gender, but also in relation to obesity and lipid metabolism disorders [13–15]. For overweight or obese patients, who usually do fewer activities, natural stone removal is more difficult, and if surgical treatment is performed, the difficulty and risk of surgery increase. Therefore, for these patients, the recurrence of kidney stone leads to secondary or multiple treatments that not only pose a greater risk to the patient's health but also increase the difficulty and cost of kidney stone treatment and increase the medical burden on society, making it more difficult and necessary to prevent stone recurrence in these patients.

One of the reasons for the high recurrence rate of kidney stone in obese or overweight patients is due to insufficient awareness of prevention. Therefore, it is very meaningful to find relevant indicators to make a preliminary prediction about whether patients are at high risk of recurrence, which can focus on strengthening health education and dietary intervention for high-risk patients, and better prevent recurrence of kidney stone. At present, there are few international studies on the risk indicators of kidney stone recurrence in overweight and obese patients. In this study, 301 overweight and obese patients hospitalized at the First Affiliated Hospital of Anhui Medical University from March 2016 to March 2022 were involved and divided into stone recurrence group and non-recurrence group, and relevant parameters were collected for statistical study to find statistically different indicators and to explore their predictive value.

## Materials and methods

### Study population and date acquisition

After the ethics committee of the First Affiliated Hospital of Anhui Medical University approval (Number: Quick-PJ2023-01–12), we retrospectively analyzed the clinical data of 768 patients with kidney stone who underwent surgical treatment in the department of urology, the First Affiliated Hospital of Anhui Medical University from March 2016 to March 2022. We calculated the BMI of these kidney stone patients and selected those with BMI ≥ 25 kg/m^2^ (overweight or obesity) as the study subjects [16]. Age, gender, BMI, albumin (ALB), globulin (GLOB), direct bilirubin (DBIT), indirect bilirubin (IBIL), ALT, AST, urea, creatinine (Cr), cystatin C (Cys C), eGFR, HDL-C, VLDL, LDL-C, Apo A, Apo B, lipoprotein, serum potassium (K^+^), serum sodium (Na^+^), serum chloride (Cl^−^), serum calcium (Ca^2+^), serum phosphorus (P), serum magnesium (Mg^2+^), blood glucose (GLU), glycated serum protein (GSP), neutrophils (NE), lymphocytes (LYM), red blood cells (RBC), hemoglobin (Hb), platelets (PLT), and history of hypertension and hyperglycemia were collected.

Inclusion criteria: ① BMI ≥ 25 kg/m^2^; ② Patients with kidney stone; ③ Adults, who age ≥ 18 years old; Exclusion criteria: ① Patients with incomplete electronic medical record information; ② Patients with severe cardiovascular diseases, brain diseases, lung diseases, and malignancy history; ③ Patients younger than 18 years old and adult patients with a BMI < 25 kg/m^2^.

### Parameters detection methods and acquisition sources

The patient's blood parameters such as red blood cells (RBC), hemoglobin(Hb), platelets (PLT), neutrophils(NE), lymphocytes(LYM) were tested by a Automatic Flow Cytometer, and the albumin, globulin, direct bilirubin, indirect bilirubin, ALT, AST, urea, creatinine, cystatin C, eGFR, HDL-C, VLDL, LDL-C, Apo A, Apo B, lipoprotein, serum potassium, serum sodium, serum chloride, serum calcium, serum phosphorus, serum magnesium, blood glucose, glycated serum protein were tested by a Roche Cobas 6000 chemistry analyzer from venous blood drawn before the patient's hospitalization for surgery. We obtained the relevant data by reviewing the patient's previous inspection reports, and the patient's age, gender, hypertension, and hyperglycemia history were obtained by reviewing the patient's electronic medical record.

### Definition of kidney stone recurrence and follow-up protocol

We evaluated patients for stone recurrence by asking if they had a previous history of stones and by reviewing previous and current imaging data. We followed up the patients for six year and instructed them to complete KUB, CT, or abdominal ultrasound to determine whether they had a stone recurrence. Stone recurrence was defined as a new stone that was not previously present, and residual stones were excluded.

### Ae index calculation and definition

Apo B is an apolipoprotein and eGFR is the estimated glomerular filtration rate, they were all tested by an automated biochemical analyzer. eGFR was calculated by CKD-EPI or modified CKD-EPI formula based on the creatinine level in venous blood combined with age, sex, body weight, and cystatin C level, it is a better indicator of the renal function status [17]. Ae index was obtained by the ratio of Apo B to eGFR and multiply by 1000 (Apo B × 1000/eGFR), in order to estimate the risk of kidney stone recurrence in people with BMI ≥ 25 kg/m^2^ in this study.

### Statistical methods

Statistical analysis was performed using the R software (Version 4.2.2, https://cran.r-project.org/bin/windows/base/) and Empower software (https://www.empowerstats.net/cn/; X&Y Solutions, Inc., Boston, MA, USA). Firstly, we used the Kolmogorov-Smirnova test to do the normality test for the measurement. For those conforming to normal distribution, we denoted it by x ± s and the differences between the two groups were used the independent samples t-test; those conforming to skewed distribution were expressed as M (P_25_, P_75_) and the differences between the two groups were used Wilcoxon rank-sum test. Then, we performed univariate and multivariate logistic regression analysis of the screened statistically significant parameters. Lastly, we used R software package “pROC” to plot the ROC curve to estimate the prediction accuracy of the selected parameters, and used a smooth curve to evaluate the relationship between Ae index and kidney stone recurrence [18]. Differences were considered statistically significant at *p* < 0.05.

## Results

### Patients clinical characteristics

**Fig. 1:**
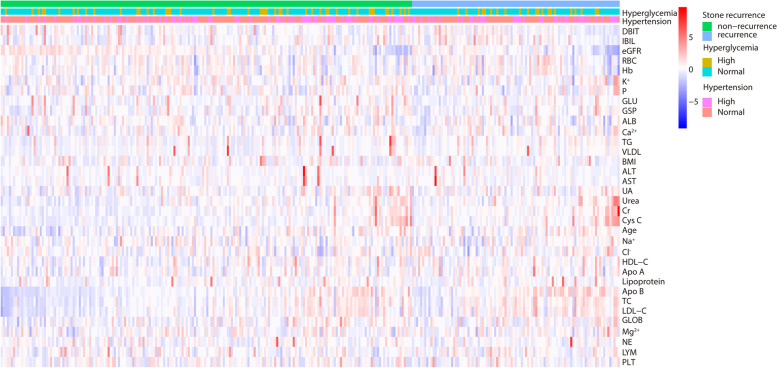
The heat map suggests abnormal expression of parameters such as creatinine, urea, blood lipids, and others in the stone recurrence group

**Fig. 2:**
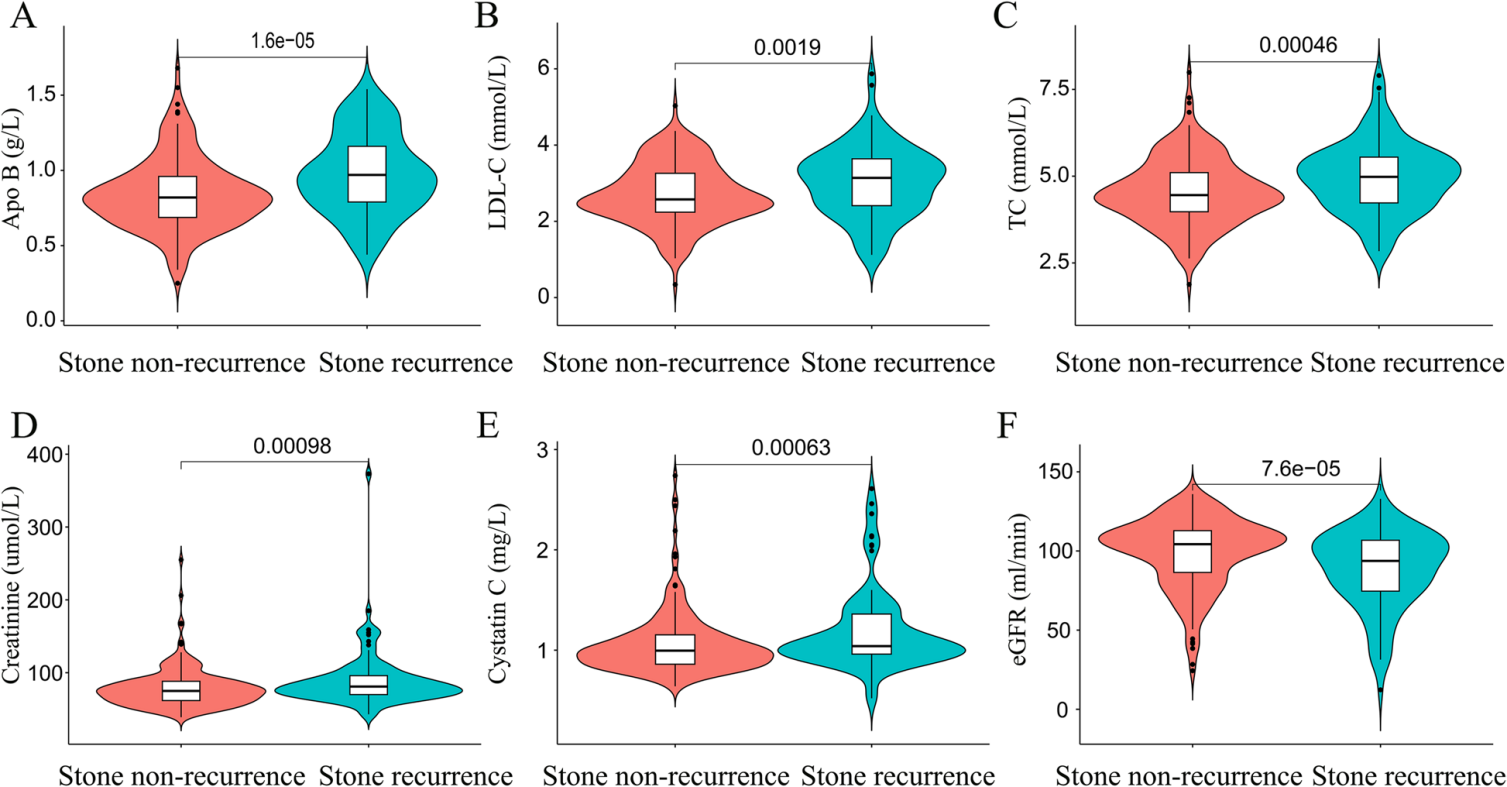
**A-E**. The results of the study revealed that Apo B, LDL-C, TC, creatinine, and cystatin C expressions were higher in the kidney stone recurrence group than non-recurrence group; **F**. eGFR values were lower in the kidney stone recurrence group compared to non-recurrence group. *P* < 0.05 as statistically different

Among these 768 patients, there were 301 patients with BMI ≥ 25 kg/m^2^, of whom 101(33.6%) had recurrence of stones, including 61(60.4%) cases of male and 40(39.6%) cases of female. The routine blood, biochemical tests, and glycated serum protein lab reports of patients were queried, and related parameters of age, gender, BMI, ALB, GLOB, DBIT, IBIL, ALT, AST, urea, Cr, cystatin C, eGFR, HDL-C, VLDL, LDL-C, Apo A, Apo B, lipoprotein, K^+^, Na^+^, Cl^−^, Ca^2+^, P, Mg^2+^, GLU, GSP, NE, LYM, RBC, Hb, and PLT were collected. We divided the patients into kidney stone recurrence and non-recurrence groups and drew heat maps based on their clinical parameters (as shown in Fig. 1). The heat map suggests abnormal expression of parameters such as creatinine, urea, blood lipids, and others in the kidney stone recurrence group. In order to further investigate the differences in the relevant parameters between the two groups, we conducted univariate analysis of the two groups of data, for which we used the Chi-square test for the count data, and we used the independent samples t-test for the measurement data that conformed to a normal distribution, and the Wilcoxon rank-sum test for those that did not conform to a normal distribution (as shown in Table S1). The results of the study revealed that creatinine, cystatin C, TC, LDL-C, and Apo B expressions were higher in the kidney stone recurrence group than non-recurrence group, and eGFR values were lower than in the stone non-recurrence group (as shown in Fig. 2).

### Logistic regression analysis

Then univariate and multivariate logistic analyses were performed for the parameters with differences screened above, such as creatinine, cystatin C, eGFR, LDL-C, Apo B, and TC (as shown in Table 1). Univariate logistic analysis revealed that creatinine, cystatin C, Apo B, LDL-C, and TC were high risk factors for kidney stone recurrence among those with BMI ≥ 25 kg/m^2^, while eGFR was a protective factor. Then we performed multivariate logistic analysis, and the most differentially influential factors were found to be Apo B and eGFR. And the results of the study found that each unit increase in Apo B was associated with an 11.028-fold increase in the probability of stone recurrence, while the risk of kidney stone recurrence decreased by 2.4% for each unit increase in eGFR.

**Table 1:** Univariate and multivariate logistic analysis of  kidney stone recurrence

Variable	Univariate analysis			Multivariate analysis		
	Wals	Sig	OR(95%CI)	Wals	Sig	OR(95% CI)
Apo B	17.486	< 0.001	8.376 (3.093–22.680)	20.663	< 0.001	11.028 (3.917–31.047)
eGFR	13.703	< 0.001	0.980 (0.970–0.991)	16.984	< 0.001	0.976 (0.965–0.988)
Creatinine	7.306	0.007	1.012 (1.003–1.021)			
Cystatin C	8.097	0.004	2.747 (1.369–5.508)			
LDL-C	9.423	0.002	1.588 (1.182–2.134)			
TC	11.266	0.001	1.543 (1.198–1.988)			

### Ae index’s smoothing curve, threshold effect, and subgroup analysis on kidney stone recurrence

As mentioned above, the expression level of Apo B was positively correlated with kidney stone recurrence and the expression level of eGFR was negatively correlated with kidney stone recurrence, we considered combining the two aspects to improve the sensitivity and specificity of the prediction of kidney stone recurrence. Then we proposed the Ae index, which was obtained by the ratio of Apo B to eGFR and multiplied by 1000, in order to predict the probability of kidney stone recurrence.

**Fig. 3:**
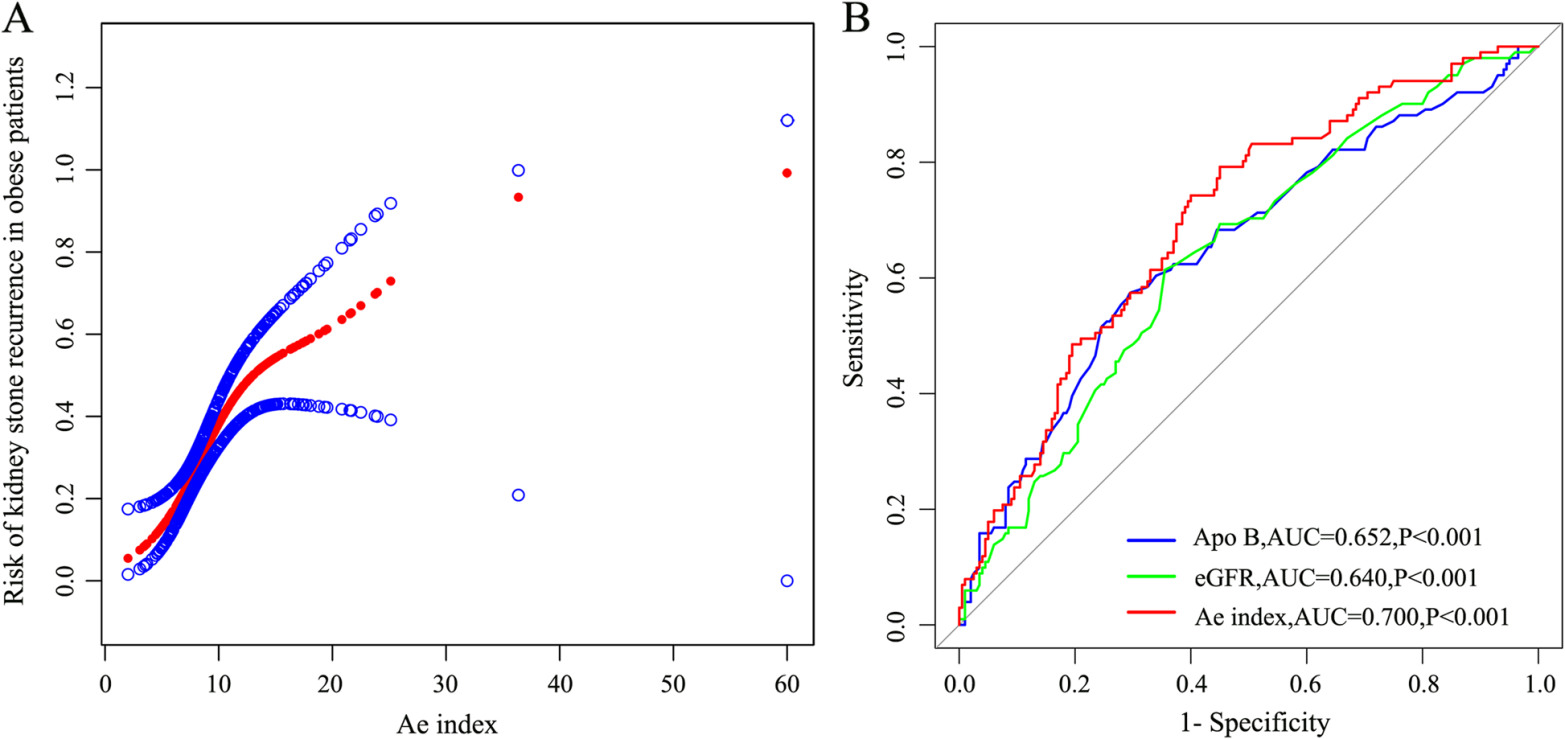
**A** The relationship between Ae index and kidney stone recurrence was detected by smoothed curve fitting, and a non-linear relationship was found between them with an inflection point. The area between two blue lines is 95% CI. Each point of the red line represents an Ae index. **B** We plotted the ROC curves of Apo B, eGFR, and Ae index, respectively, and found the prediction accuracy of the Ae index was higher than that of Apo B and eGFR alone

A smoothed curve fitting model have been used to analyze the relationship between Ae index and kidney stone recurrence. The results demonstrated a non-linear relationship between the Ae index and kidney stone recurrence (as shown in Fig. 3A and Table 2). Based on a two-segment linear regression model, we calculated the Ae index inflection point to be 9.16, and the OR on the left side of the inflection point was 1.574 (95% CI: 1.228–2.018), which means that for every 1 unit increase in Ae index, the risk of kidney stone recurrence increased by 57.4%. Whereas the OR on the right side of the inflection point was 1.088 (95% CI: 1.007–1.177), which means that for every 1 unit increase in Ae index, the risk of kidney stone recurrence increased by 8.8%. Then we performed logistic regression analysis on the Ae index and divided the Ae index into three intervals according to the tertiles method for subgroup analysis to investigate the relationship between the index and kidney stone recurrence in each interval, and found the higher the Ae index, the greater the risk of recurrence of kidney stone (as shown in Table 2).

**Table 2:** Analysis of Ae index with kidney stone recurrence

Ae index	ULR Test OR (95%CI)	PLR Test OR (95%CI)	Logistic analysis OR (95%CI)	*P*	LRT test *P* value
Total	1.176 (1.100,1.257)			< 0.0001^a^	0.010^c^
Inflection point					
Ae ≤ 9.16		1.574 (1.228,2.018)		0.0003^b^	
Ae > 9.16		1.088 (1.007,1.177)		0.0336^b^	
Subgroup					
2.01 < Ae ≤ 7.72			1	< 0.001^d^	
7.72 < Ae ≤ 10.77			2.586 (1.313,5.093)	0.006^d^	
10.77 < Ae ≤ 60.00			5.571 (2.874,10.800)	< 0.001^d^	

^a^ULR, Univariate linear regression

^b^PLR, Piecewise linear regression

^c^LRT, Logarithmic likelihood ratio

^d^Logistic regression analysis; statistically significant

*p* < 0.05

### ROC curves drawing and prediction of kidney stone recurrence

To clarify the prediction accuracy of Ae index, Apo B, and eGFR for kidney stone recurrence, we used the R software package “pROC” to draw ROC curves to assess their sensitivity and specificity, respectively (as shown in Fig. 3B). And we found the prediction accuracy of the Ae index is higher than that of Apo B and eGFR alone (as shown in Table 3).

**Table 3:** Sensitivity and specificity of univariate and Ae index

Category	Cut-off	Sensitivity (%)	95%CI	Specificity (%)	95% CI	+ LR	-LR
Apo B	> 0.91 g/L	57.43	47.2–67.2	70.50	63.7–76.7	1.95	0.60
eGFR	≤ 98 ml/min	61.39	51.2–70.9	64.50	57.4–71.10	1.73	0.60
Ae	> 8.72	74.26	64.6–82.4	60.00	52.9–66.80	1.86	0.43

**Fig. 4:**
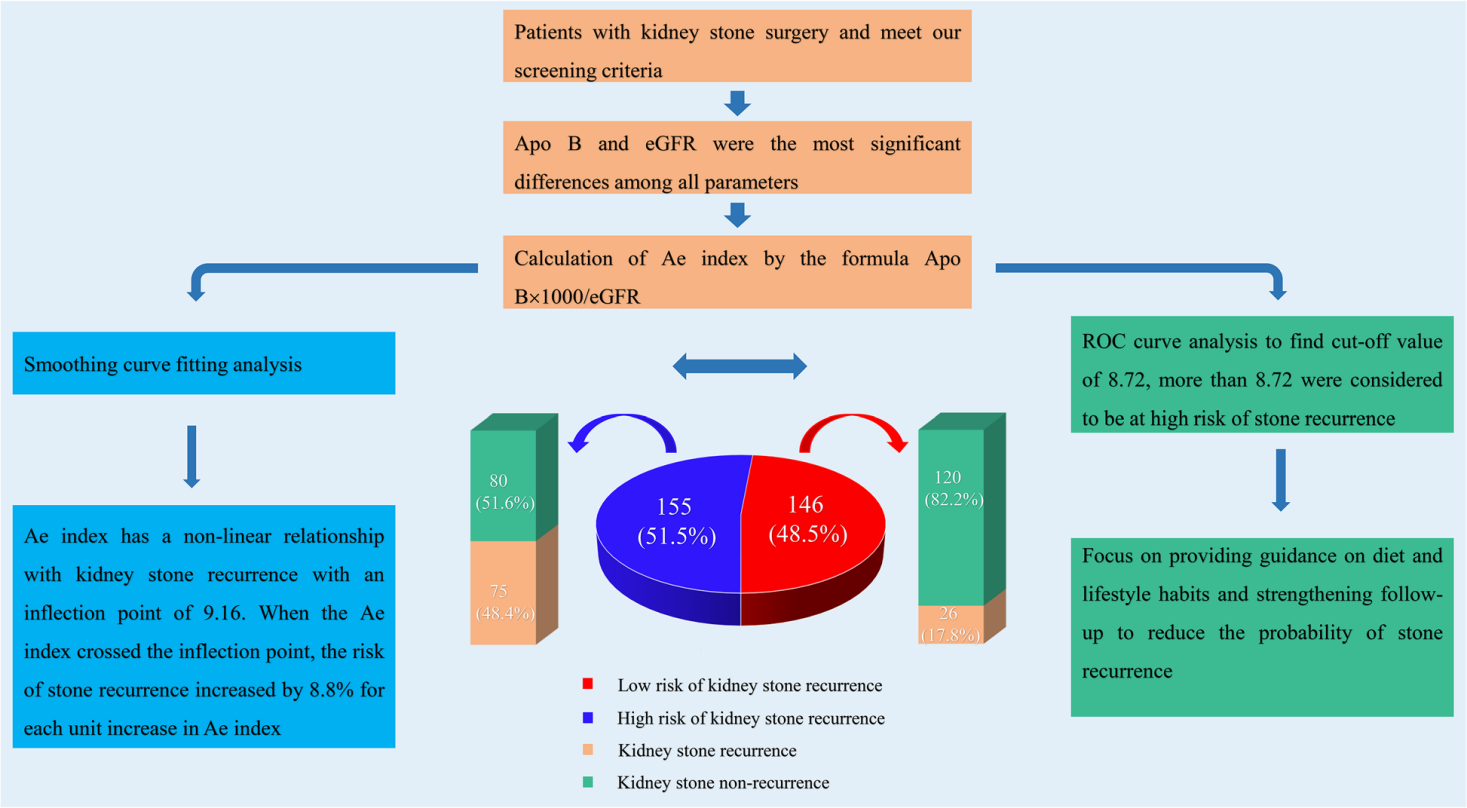
Flow chart of Apo B and eGFR parameters screening and Ae index analysis

In summary, the most significant variables Apo B and eGFR affecting kidney stone recurrence in overweight and obese patients were screened by univariate and multifactor analysis, and the formula Apo B × 1000/eGFR was used to calculate the Ae index. By smoothing curve fitting analysis, we found a non-linear relationship between Ae index and kidney stone recurrence, ROC curve analyses suggested the predictive accuracy of Ae index higher than any single variable (as shown in Fig. 4).

## Discussion

In this study, we divided the kidney stone patients into stone recurrence and non-recurrence group, relevant clinical parameters of the patients were collected for statistical analysis, and it was found that Apo B and eGFR were the most significant factors affecting stone recurrence. Apo B is the main structural protein of low-density lipoprotein cholesterol (LDL-C), which reflects the expression level of LDL-C, and it is present on the surface of LDL, and cells generally take up LDL-C by recognizing Apo B [19]. Elevated Apo B reflects lipid disorders in the body and has been reported to be closely related to the development of coronary heart disease [20]. eGFR is the estimated glomerular filtration rate, which assesses the kidney's ability to remove metabolic wastes from the body by measuring the glomerular capacity to remove creatinine per minute, and is an indicator of kidney function [21]. The normal value of eGFR is 110-130 ml/min, which is negatively correlated with the degree of renal damage [22]. The decrease of eGFR indicates that renal function is impaired, and the more obvious the decrease, the more serious the renal damage [23].

Recurrence of kidney stone is mainly caused by supersaturated precipitation of crystals in the urine in the kidney, which is closely related not only to the patient's personal basical disease, hypercalcemia, and race, but also to the patient's metabolic abnormalities [14, 24, 25]. Some studies have shown that hypertension and hyperglycemia are positively associated with the recurrence of kidney stone, and abnormal lipid metabolism is also closely associated with the recurrence of kidney stone [26–28]. Impaired kidney function means the kidney's ability to urinate is diminished, mainly in the form of slower urinary excretion, resulting in the easy supersaturated precipitation of crystals in the urine, which in turn leads to the recurrence of stone. Therefore, kidney stone recurrence rate was high in those with low eGFR values, and it was negatively correlated with stone recurrence. In addition, elevated Apo B indicates increased LDL cholesterol (LDL-C) expression and excessive LDL-C deposition on the glomerular vessel wall, leading to glomerular vascular sclerosis and stenosis, affecting kidney function and increasing the likelihood of stone recurrence.

Ae index is closely related to Apo B and eGFR expression and is associated with the recurrence of kidney stone. The relationship between Ae index and kidney stone recurrence was detected by smoothed curve fitting, and a non-linear relationship was found between them with an inflection point was 9.16, and the increase in the risk of kidney stone recurrence slowed down as the Ae index increased after crossing the inflection point. Then we drew the ROC curve and found that its predictive accuracy was higher than that of Apo B and eGFR alone. The Ae index combines the advantages of Apo B and eGFR to improve the predictive ability of kidney stone recurrence, which is helpful to identify patients with high risk of stone recurrence. Then we focus on health education for them, reminding low salt and low protein diet, and instructing them to consume calcium, potassium, magnesium, and vit B6-rich vegetables and fruits in moderation, and increasing the frequency of review to reduce the possibility of recurrence.

This study proposes a new index named Ae index and it can predict the probability of stone recurrence in overweight or obese people. Its advantages are shown in the following two aspects: Firstly, the index is simple to calculate, and the Apo B and eGFR parameters involved can be obtained by biochemical assays, it can be tested in most primary care hospitals at a lower medical cost, convenient and economical, saves patient time and reduces medical burden. Secondly, the ROC curve analysis suggested that people with Ae index higher than 8.72 were at high risk of kidney stone recurrence, and smoothed curve revealed the risk of kidney stone recurrence increased by 8.8% for each unit increase when the index across the inflection point of 9.16. We used the Ae index to briefly assess postoperative stone patient, and for those at high risk, we need to focus on strengthening health education, enhancing diet guidance, and increasing the frequency of follow-up to reduce the probability of recurrence. At the same time, this study has the following shortcomings: Firstly, this study is a retrospective study, the number of included study subjects are small, some information data are missing, there is a certain selection bias, and the sample size needs to be further expanded in the future. Secondly, although the specificity and sensitivity of the Ae index was higher than any other univariate, it was still not particularly significant, and collection of more parameters to construct predictive models to improve the sensitivity and specificity of the prediction of kidney stone recurrence could be considered in the future. Thirdly, the index is currently limited to overweight and obese people with kidney stone, and further studies are needed to expand the participating population. Fourthly, this study is a single-center study, and further multicenter studies are needed in the future.

## Conclusion

This study proposes a new Ae index to assess the risk of stone recurrence in overweight and obese kidney stone patients, which has a non-linear relationship with kidney stone recurrence. Patients with Ae index over 8.72 are at high risk for stone recurrence, and the risk of kidney stone recurrence increased by about 8.8% for each unit increase in Ae index.

### Supplementary Information

**Additional file 1: Table S1.** Univariate analysis of patient data of overweight and obesity stone recurrence.

## Data Availability

Data is available with permission from the corresponding author.
