# Supplementary material for: Ae index is an independent predictor of kidney stone recurrence in overweight and obese patients
Source: BMC Urol. 2023 Sep 23;23:151. doi: 10.1186/s12894-023-01321-7 (PMC10518111; doi:10.1186/s12894-023-01321-7)
Supplement: Supplementary file 1 — Additional file 1: Table S1. Univariate analysis of patient data of overweight and obesity stone recurrence. [file 12894_2023_1321_MOESM1_ESM.docx]

Table S1. Univariate analysis of patient data of overweight and obesity stone recurrence

| Categories | Stone recurrence(%) | Stone non-recurence(%) | χ^2^/t/Z | P |
| --- | --- | --- | --- | --- |
| Total | 101(33.6%) | 200(66.4%) |  |  |
| Gender |  |  | 0.022 | 0.881^a^ |
| male | 61(33.9) | 119(66.1) |  |  |
| female | 40(33.1) | 81(66.9) |  |  |
| Hypertension |  |  | 0.096 | 0.757^a^ |
| Yes | 37(34.6) | 70(65.4) |  |  |
| No | 64(33.0) | 130(67.0) |  |  |
| Hyperglycaemia |  |  | 0.391 | 0.532^a^ |
| Yes | 21(30.4) | 48(69.6) |  |  |
| No | 80(34.5) | 152(65.5) |  |  |
| Age (years) | 51(44,56) | 48.5(41,54) | -1.789 | 0.074^c^ |
| BMI(kg/m^2^) | 27.57±2.31 | 27.65±2.37 | -0.303 | 0.762^b^ |
| Albumin(g/L) | 44.33±3.73 | 44.78±3.20 | -1.073 | 0.284^b^ |
| Globulin(g/L) | 26.73±4.30 | 26.39±4.10 | 0.672 | 0.502^b^ |
| Direct bilirubin (umol/L) | 3.96±1.60 | 4.17±1.92 | -0.912 | 0.363^b^ |
| Indirect bilirubin(umol/L) | 8.76±3.52 | 8.32±3.40 | 1.043 | 0.298^b^ |
| ALT(u/L) | 27.63±25.94 | 29.65±27.54 | -0.610 | 0.542^b^ |
| AST(u/L) | 21.22±12.95 | 22.18±12.52 | -0.619 | 0.436^b^ |
| Urea(mmol/L) | 5.51(4.55,6.45) | 5.24(4.27,6.20) | -1.789 | 0.074^c^ |
| Creatinine(umol/L) | 81(70.00,96.50) | 75(61.25,88.75) | -3.296 | <0.001^c^ |
| Uric acid(umol/L) | 332(280,401) | 335(276.25,412.25) | -0.090 | 0.928^c^ |
| Cystatin C (mg/L) | 1.04(0.96,1.37) | 0.99(0.86,1.16) | -3.421 | < 0.001^c^ |
| eGFR(ml/min) | 94(74,107) | 104.5(86.25,113.00) | -3.957 | <0.001^c^ |
| TC (mmol/L) | 4.98(4.22,5.55) | 4.46(3.95,5.11) | 3.494 | <0.001^c^ |
| TG (mmol/L) | 2.01±0.88 | 1.90±1.20 | 0.762 | 0.447^b^ |
| HDL-C (mmol/L) | 1.18±0.29 | 1.13±0.30 | 1.225 | 0.222^b^ |
| VLDL (mmol/L) | 0.78±0.45 | 0.75±0.62 | 0.326 | 0.745^b^ |
| LDL-C (mmol/L) | 3.14(2.41,3.65) | 2.58(2.24,3.26) | -3.105 | 0.002^c^ |
| Apo A (g/L) | 1.48±0.27 | 1.43±0.23 | 1.632 | 0.104^b^ |
| Apo B (g/L) | 0.97(0.79,1.18) | 0.82(0.68,0.96) | -4.320 | <0.001^c^ |
| Lipoprotein (mmol/L) | 184(73.5,312.5) | 153.5(61.5,290.5) | -0.819 | 0.413^c^ |
| K^+^(mmol/L) | 3.97±0.43 | 3.99±0.35 | -0.477 | 0.634^b^ |
| Na^+^(mmol/L) | 140.60±2.26 | 140.74±2.22 | -0.510 | 0.610^b^ |
| Cl^-^(mmol/L) | 102.70±3.00 | 102.45±2.68 | 0.723 | 0.470^b^ |
| Ca^2+^(mmol/L) | 2.35±0.12 | 2.35±0.12 | 0.128 | 0.898^b^ |
| P (mmol/L) | 1.07±0.22 | 1.10±0.20 | -1.143 | 0.254^b^ |
| Mg^2+^(mmol/L) | 0.87±0.08 | 0.86±0.08 | 0.357 | 0.721^b^ |
| Glu(mmol/L) | 5.32(4.85,5.88) | 5.37(4.89,6.00) | -0.881 | 0.378^c^ |
| GSP(umol/L) | 250(229.5,266.5) | 249.00(232.00,278.00) | -0.488 | 0.625^c^ |
| Neutrophil (10^9^/L) | 3.88±1.67 | 4.04±1.56 | -0.811 | 0.418^b^ |
| Lymphocyte (10^9^/L) | 2.01±0.61 | 1.94±0.64 | 0.948 | 0.344^b^ |
| RBC(10^12^/L) | 4.67±0.56 | 4.68±0.49 | -0.102 | 0.919^b^ |
| Hb(g/L) | 136.93±19.36 | 137.60±16.12 | -0.317 | 0.751^b^ |
| PLT(10^9^/L) | 222.74±63.82 | 226.90±63.70 | -0.534 | 0.594^b^ |

a. Chi-squared; b. independent samples t-test; c. Wilcoxon rank-sum test.

statistically significant: p < 0.05.
